# Dr. William F. H. Montgomery

**Published:** 1860-03

**Authors:** 


### NECROLOGICAL NOTICES.

DR. WILLIAM F. H. MONTGOMERY.—It is with the deepest regret that we are called upon to record the name of this distinguished member of our profession among those of the illustrious dead. Professor Montgomery, well known as an elegant lecturer and copious author, died in Dublin, at the age of sixty-two, of disease of the heart, on the 21st of December, 1859. Early in life he evinced talents of the highest order, and obtained a scholarship in Trinity College, in 1820. Five years subsequently he took the degree of Bachelor of Medicine, and soon after entering upon his professional career he gave lectures on midwifery to full classes of students, and to his exertions was due the founding of the Chair of Midwifery in the King's and Queen's College of Physicians in Ireland, which he filled with so much distinction for a period of thirty years.

Dr. Montgomery's contributions to medical and obstetrical literature were very numerous, and are to be found chiefly in the *Dublin Quarterly Journal.* He also wrote several important articles for the Cyclopedia of Practical Medicine; but his reputation is based mainly upon his "Exposition of the Signs and Symptoms of Pregnancy," and on his essay upon "Spontaneous amputation of the fœtal limbs in utero."

